# Gastrointestinal Dysbiosis in Neuro-Critically Ill Patients: A Systematic Review of Case-Control Studies

**DOI:** 10.7759/cureus.50923

**Published:** 2023-12-21

**Authors:** Haseeb Mehmood Qadri, Saad Abdullah Dar, Raahim A Bashir, Manal Khan, Salamat Ali, Abdul Subhan Zahid, Asim Ali, Marriam, Saba Waheed, Maha Saeed

**Affiliations:** 1 Neurosurgery, Unit-I, Punjab Institute of Neurosciences, Lahore, PAK; 2 Surgery, Allama Iqbal Medical College, Lahore, PAK; 3 Neurological Surgery, CMH Lahore Medical College and Institute of Dentistry, Lahore, PAK; 4 Surgery, Nawaz Shareef Medical College, Gujrat, PAK; 5 Surgery, Ameer Ud Deen Medical College, Lahore, PAK; 6 General Surgery, Lahore General Hospital, Lahore, PAK; 7 Surgery, Independent Medical College, Faisalabad, PAK; 8 Emergency Medicine, Akhtar Saeed Medical and Dental College, Lahore, PAK; 9 Internal Medicine, Akhtar Saeed Medical and Dental College, Lahore, PAK

**Keywords:** brain tumor, dysbiosis, neurocritical, gut flora, gut microbiota

## Abstract

The human gastrointestinal tract (GIT) has a rich and pre-programmed microbiome. This microbiome is essential for physiological functions such as digestion, immunity, metabolism, and structural integrity, and of prime concern to us in conducting this study is the nervous system communication. This two-way communication between the GIT and central nervous system (CNS) is known as the gut-brain axis (GBA) and has implications for neurocritical disease. A change in any factor relating to this microbiome is known as gut dysbiosis; this can lead to aberrant communication through the GBA and in turn, can contribute to disease states. The primary objective of this study is to determine the cause-specific dysbiotic organisms in neuro-critically ill patients and their effects.

We performed this study by searching published literature as per Preferred Reporting Items for Systematic Reviews and Meta-Analyses (PRISMA) guidelines. Studies that defined gastrointestinal dysbiosis in neuro-critically ill patients were retrieved using Boolean search from 2000 to 2023 via PubMed and Google Scholar and narrowed the results down to five prospective case-control studies. We performed their quality assessment. The results concluded that in neurocritical illnesses such as encephalitis, brain tumors, intracerebral hemorrhage, and ischemic stroke, fluctuations in specific microbiota correlated with disease severity and prognosis. Moreover, the inhabiting population of dysbiotic organisms in neuro-critically ill patients were different in different diseases and there were no similarities in the composition of gut microbiota in these diseases. Taking stroke patients as an example; increased Enterobacteriaceae and lower Lachnospiraceae microbiome levels were found in patients with a higher stroke dysbiosis index (SDI). Those patients who developed stroke-associated pneumonia (SAP) displayed higher levels of Enterococcus species. In conclusion, dysbiosis has a major effect on neuro-critically ill patients’ disease states and dysbiotic organisms can be used as a biomarker for disease. Further prospective studies on this topic are warranted for potential neurological and prognostic correlations.

## Introduction and background

The connection between the gastrointestinal tract (GIT) and the central nervous system (CNS) is referred to as the “gut-brain axis” (GBA) [1]. The complex link between GIT and CNS involves the enteric nervous system (ENS), sympathetic and parasympathetic branches of the autonomic nervous system (ANS), as well as pathways for neuro-immune and neuroendocrine signaling [1]. Changes in the composition, quantity, and activity of the gut microbiome led to disrupted communication between the gut and the brain. This disruption has been recognized as a significant contributing factor to various diseases including those affecting the brain. When the homeostasis of this connection is altered, it can lead to changes in the intestinal barrier resulting in the leakage of toxins into the bloodstream, inflammation throughout the body, and a higher risk of infection [2].

The co-occurrence of gastrointestinal and psychiatric disorders suggests that the “gut-brain axis” is a potential contributor to the pathogenesis of these disorders [3]. Other than diet and inflammatory conditions affecting GIT, conditions involving the brain and spinal cord can also cause dysbiosis [4,5]. The gut microbiota plays a significant role in producing neurotransmitters, like serotonin which are key players in the regulation of mood and behavior [3]. Dysbiosis can disrupt serotonin production leading to depression and anxiety disorders. It can also trigger systemic inflammation, which is associated with the neuroinflammation observed in Alzheimer's disease and can also contribute to immune system dysfunction which leads to the advancement of multiple sclerosis (MS) [6]. The immuno-inflammatory pathways in the GBA are involved in the pathogenesis of epilepsy, secondary to dysbiosis [7]. Furthermore, bacterial translocation and raised systemic inflammatory markers due to gut dysbiosis are related to the severity of schizophrenia [3].

It is believed that the digestive tract is the main organ of the immune response. Increasing data indicates that intestinal microflora is an important factor in the development, sequelae, and treatment of stroke [8]. An evidence-based study showed that an antibiotic-specific shift in the microbiome composition in the gut is linked with neuroprotection in stroke patients [9]. Moreover, the degree of gut dysbiosis can serve as a diagnostic indicator for determining the severity of traumatic brain injury and providing valuable insights for guiding treatment decisions [5]. Hence, the gut microbiota can be used as a therapeutic target to treat various diseases [5]. Taking steps to improve gut health, such as using probiotics and making dietary changes, may show promising outcomes in managing and potentially preventing these conditions [10]. There is an absence of cumulative evidence on gut dysbiosis in neuro-critically ill patients. Hence, we attempt to combine the available literature under this systematic review.

## Review

Objectives

The objectives of this review article were set to determine the similarities and differences in inhabiting a population of dysbiotic organisms in neuro-critically ill patients; to evaluate cause-specific dysbiotic flora and to establish the specific organisms or cytokines as the biomarker of diseases.

Methodology

**Figure 1:**
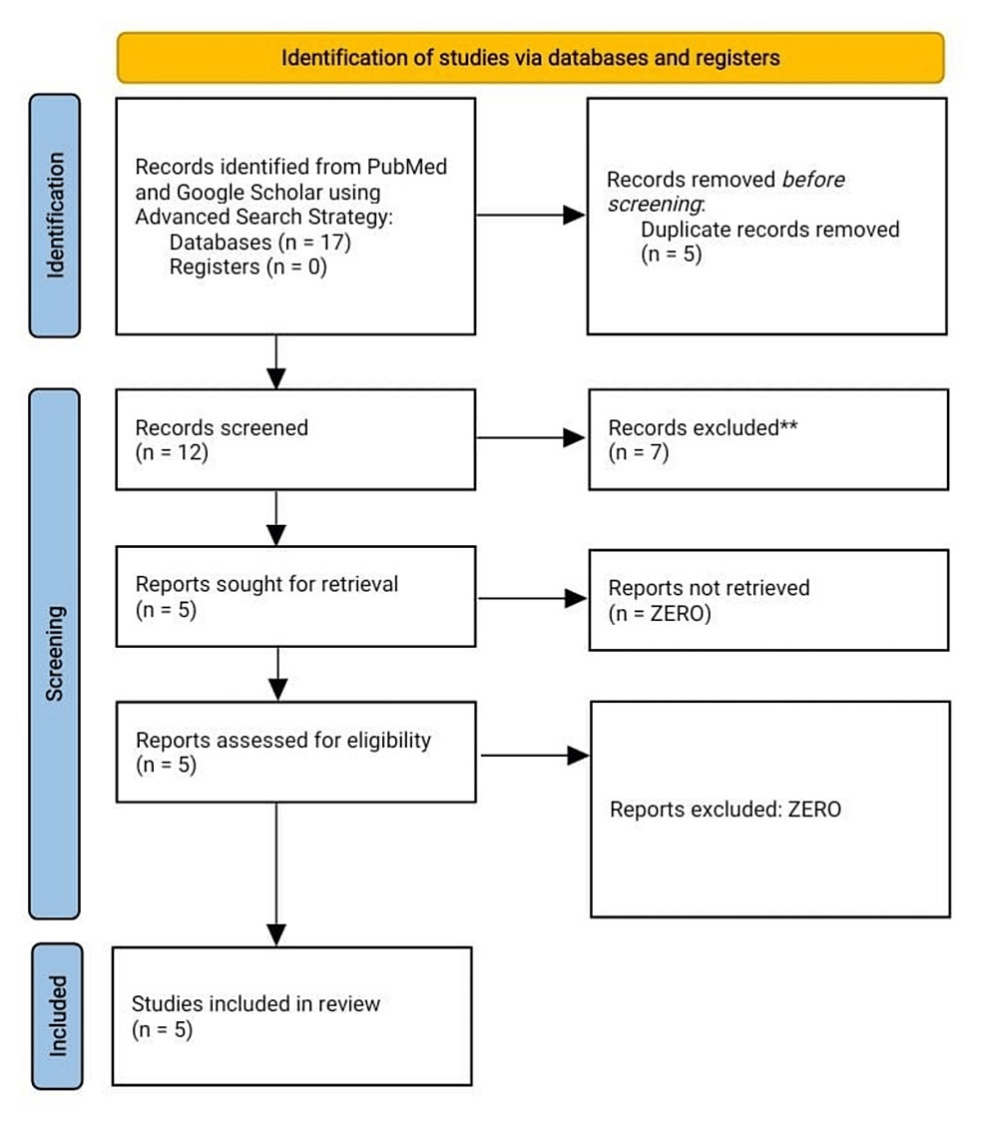
Preferred Reporting Items for Systematic Reviews and Meta-Analyses (PRISMA) flow sheet.

We conducted a systematic review on the topic of gastrointestinal dysbiosis in neurocritical patients as per the guidelines of Preferred Reporting Items for Systematic Reviews and Meta-Analyses (PRISMA). Case-control studies, randomized control trials, and cohort studies in the English language on neurocritical patients which were open access, published between 2000 and 2023 were included in our study. Moreover, non-English language studies, letters to editors, editorials, case reports, case series, clinical images, animal studies, and cadaveric studies were excluded during secondary scrutiny of existing literature. We used PubMed Central and Google Scholar to find the published original articles. The Boolean search strategy was employed as: “Microbiota” AND “Neuro critical,” “Microbiota” AND “Neurocritical,” “Gut Flora” AND “Neuro critical,” “Gut Flora” AND “Neurocritical,” “Dysbiosis” AND “Neurocritical,” “Dysbiosis” AND “Neuro Critical.” There was a clear scarcity of English scientific literature on this topic and a total of five prospective case-control studies were retrieved using this strategy (Figure 1).

The quality of the included studies was assessed using the Joanna Briggs Institute (JBI) Critical Appraisal Checklist for case-control studies (Table 1).

**Table 1:** Joanna Briggs Institute critical appraisal checklist for case control studies. Y: Yes, N: No, N/A: Not Available and U: Unclear

Sr. #	Questions	Gong et al. [11]	Li et al. [12]	Luo et al. [13]	Xu et al. [14]	Xia et al. [15]
1.	Were the groups comparable other than the presence of disease in cases or the absence of disease in controls?	Y	Y	U	Y	Y
2.	Were cases and controls matched appropriately?	Y	Y	Y	Y	Y
3.	Were the same criteria used for identification of cases and controls?	Y	U	U	Y	Y
4.	Was exposure measured in a standard, valid and reliable way?	Y	Y	Y	Y	Y
5.	Was exposure measured in the same way for cases and controls?	Y	Y	N	Y	Y
6.	Were confounding factors identified?	Y	Y	Y	Y	Y
7.	Were strategies to deal with confounding factors stated?	Y	Y	Y	Y	Y
8.	Were outcomes assessed in a standard, valid and reliable way for cases and controls?	Y	Y	Y	Y	Y
9.	Was the exposure period of interest long enough to be meaningful?	Y	N/A	Y	Y	U
10.	Was appropriate statistical analysis used?	Y	Y	Y	Y	Y
	Overall Appraisal	Included	Included	Included	Included	Included

Summary of included studies

Included studies are prospective case-control studies published in 2019 and 2022, from China. Two studies have documented the effects of cerebrovascular disorders on microbiota dysbiosis, while the other two studies have correlated brain tumors and encephalitis with dysbiosis. The total number of patients in these studies was N = 774 (Table 2) [11-15].

**Table 2:** Publication details of included studies.

Study by	Parameters			
	Title of study	Year of publication	Country of publication	Study design
Gong et al. [11]	Disturbance of gut bacteria and metabolites are associated with disease severity and predict outcome of NMDAR encephalitis: A prospective case–control study	2022	China	Prospective Case–Control Study
Li et al. [12]	Crosstalk between the gut and brain: Importance of the fecal microbiota in patient with brain tumors	2022	China	Prospective Case–Control Study
Luo et al. [13]	Gut microbiota composition reflects disease progression, severity and outcome, and dysfunctional immune responses in patients with hypertensive intracerebral hemorrhage	2022	China	Prospective Case–Control Study
Xu et al. [14]	Dysbiosis of the intestinal microbiota in neuro-critically ill patients and the risk for death	2019	China	Prospective Case–Control Study
Xia et al. [15]	Stroke dysbiosis index (SDI) in gut microbiome are associated with brain injury and prognosis of stroke	2019	China	Prospective Case–Control Study

Out of 774 neurocritical patients, 57.76% were males, followed by 42.24% females. Affected patients and healthy controls with their mean ages were defined in some studies (Table 3) [11-15].

**Table 3:** Demographic details of included studies.

Study by	Parameters			
	Cohort Size	Gender Distribution	Mean Age	Objective of Study
Gong et al. [11]	Total 107, 58 patients, 49 healthy controls	NMDAR Encephalitis Patients=36(F)/ 22(M), Control Group=31(F)/ 18(M)	NMDAR Encephalitis Patients=34.4 Control Group=32.0	To find the relationship between the intestinal microbiota, cytokines, and clinical severity in anti-N-methyl-D-aspartate receptor (NMDAR) encephalitis.
Li et al. [12]	Total 158, 101 Primary brain tumor patients, 57 Healthy controls	Brain tumors 57 F, 44 M, Healthy controls 33 F, 24 M	57	To determine the changes of the gut microbiota community in patients with brain tumors.
Luo et al. [13]	Total 133, 64 patients with intracerebral hemorrhage, 46 coronary heart disease controls, 23 healthy controls	ICH group; 38 M, 26 F CHD group; 31 M, 15 F Healthy control; 6 M, 17 F	ICH group; 59.880 CHD group; 62.390 Healthy control; 25	To analyze the alterations in gut microbiota composition and cytokine responses in patients with hypertensive intracerebral hemorrhage (ICH).
Xu et al. [14]	Total 182, 98 neuro-critically ill patients, 84 healthy controls	Neuro-critically ill patients 61(M), 37(F), Healthy controls 52 M, 32 F	58.5	To investigate the characteristics of the gut microbiome in neuro-critically ill patients and its changes after admission.
Xia et al. [15]	Total 194, 104 patients with acute ischemic stroke, 90 healthy individuals	Stroke group 78 M, 26 F, Control group 73 M, 17 F	Stroke group 59.38 Control group 56.62	To develop a gut microbiota index in patients with acute ischemic stroke.

Gong et al. reported patients did not have any difference in their metabolite profile from their healthy controls [11]. Li et al. stated that the Firmicutes/Bacteroidetes (F/B) ratio was reduced significantly in the tumor group. Fusobacteria and Proteobacteria showed a higher abundance in people with malignant brain neoplasm than with those having benign brain tumors [12].

Luo et al. document that Enterococci and *Clostridium immocuum* were heavily populating the guts of patients with stroke-associated pneumonia (SAP) and were strongly associated with the severity of ICH [13]. Xu et al. showed increases in Enterobacteriales and Enterobacteriaceae during intensive care unit stay contributed to more than 90% mortality within six months [14]. Xi et al. demonstrated that stroke dysbiosis index (SDI) was strongly related to the severity of cerebrovascular accidents (Table 4) [15].

**Table 4:** Details of gut dysbiotic organisms in neurocritical patients.

Study by	Parameters		
	Culturable organisms which were increased in diseased group	Culturable organisms which were decreased in diseased group	
Gong et al. [11]	Bifidobacterium, Enterococcus, Clostridium innocuum_group, Roseburia	Butyricicoccus, Eubacterium_hallii_ group, Lachnospiraceae_NC2004_group, Faecalibacterium, Lachnospiraceae_NK4A136_group, Prevotella	
Li et al. [12]	Fusobacteriota, Proteobacteria, Bacteroidetes Escherichia, Shigella, Fusobacterium, Sutterella, Ruminococcus gnavus group	Bifidobacterium or Lachnospira. Firmicutes, Actinobacteria Parasutterella	
Luo et al. [13]	Enterococcus, Parabacteroides, Lachnoclostridium, Acidaminococcus, Streptococcus. Enterococcus, Parabacteroides, Blautia, Lachnoclostridium, Acidaminococcus, Alistipes, Hungatella, Clostridium innocuum_group	Prevotella Roseburia, Fusobacterium	
Xu et al. [14]	Enterobacteriaceae, Porphyromonadaceae, Enterococcaceae, Verrucomicrobiaceae, Rikenellaceae, Lactobacillaceae, Proteobacteria	Firmicutes, Bacteroidetes	
Xia et al. [15]	Enterobacteriaceae, Parabacteroides	Fecalibacterium, Clostridiaceae, Lachnospira.	

Discussion

Interrelationship of Gastrointestinal Dysbiosis and Neurocritical Patients

The GBA is a two-way communication system between the brain and the gut, allowing the brain to modulate the functions of the gut and vice versa [16]. The bidirectional communication between gut and brain can involve the ENS, ANS, vagus nerve, neurotransmitters, bacterial products, the release of damage-associated molecular patterns (DAMPs) and cytokines from the injury site of the brain and the gut, as well as migration of gut inflammatory or immune cells to the brain injury site [17]. Normal gut flora consists mainly of bacteria but also viruses, fungi, and archaea residing mainly in the colon, stomach, and small intestine [5,18]. The predominant bacterial phyla include Bacteroidetes, Actinobacteria, Verrucomicrobia, and Firmicutes [9]. Alteration in the abundance or functions of these microbiota is referred to as dysbiosis and it is not clear whether this change is the cause or effect of the neurological disorders or whether it involves a combination of both [1,2].

The changes in gut microbiota can weaken the intestinal barrier by affecting the expression of tight junctions and allowing leakage of gastrointestinal contents into the bloodstream. This “leaky gut” can then allow the contents to cross the blood-brain barrier and result in neuronal injury [5,19]. This is evidenced by the fact that probiotics that alter and restore gut flora homeostasis can reduce infection rates and improve the state of consciousness in patients with severe stroke [10]. Also, fecal microbiota transplantation can normalize post-stroke dysbiosis and is associated with an improved stroke outcome, suggesting that the restoration of gut microbiota could be a new addition to the treatment of stroke patients [20]. The CNS can influence the gut flora either by altering the ANS’s activity or by releasing endocrine mediators like catecholamines that engage with microbial receptors [19]. Alternatively, gut microbiota can influence brain function by either affecting the synthesis or degradation of neurotransmitters like serotonin, dopamine, glutamate, or gamma-aminobutyric acid (GABA). Production of microbial products, like short-chain fatty acids (SCFAs) and lipopolysaccharides (LPS) is also altered. SCFA and LPS exert their effects by activation of local and systemic immune responses [1,19,21,22]. The increased blood levels of LPS cause neuronal inflammation, promote brain edema, and favor poor outcomes after stroke [8].

The SCFAs are produced by the degradation of dietary components by the intestinal bacteria and include butyrate, acetate, and propionate [23]. Alteration of gut flora causes a reduction in the production of these SCFAs. The SCFAs can reach the blood permeate across the BBB and exert their effects on brain structure and function [7,23]. In animal models, supplementation with butyrate reduced the levels of pro-inflammatory cytokines and raised the levels of anti-inflammatory markers. Dietary supplementation with SCFAs, particularly butyric acid, significantly improves stroke outcomes by increasing the abundance of lactobacilli and reducing intestinal mucosal permeability [24].

Dysbiosis induces pro-inflammatory changes that are shown to be involved in the development of diabetes and cardiovascular diseases [25]. Various studies have found that in critically ill patients, there is a shift of gut microbiota towards pathogenic flora after only a few hours in the intensive care unit [26]. There is decreased diversity and increased abundance of Bacteroidetes after a stroke attack in most patients [27]. In neuro-critically ill patients, this change in microbiota has an influence on the mortality rates of these patients [28]. A clinical study has demonstrated that patients with large-artery atherosclerotic strokes or transient ischemic attacks have a gut microbiota composition that is distinct from the control group [2]. There was an increased number of opportunistic pathogens (e.g., Megasphaera, Enterobacter, Desulfovibrio, and Oscillibacter) and a reduced number of beneficial genera (e.g. Prevotella, Bacteroides, and Faecalibacterium) in these patients [2].

Gastrointestinal Dysbiosis and Encephalitis

In our review, the study conducted by Gong et al. on NMDAR encephalitis patients showed an increased abundance of organisms like Bifidobacterium, Enterococcus, *Clostridium innocuum* group, Roseburia and decreased abundance of Butyricicoccus, Eubacterium hallii group, Lachnospiraceae_NC2004_group, Faecalibacterium, Lachnospiraceae_NK4A136_group, and Prevotella in the patient group as compared to control group [11]. The findings of this study were in line with those of Xu et al. who showed an increase in Oscillospirales and Enterococcus and a decrease in bacteria such as Lachnospiraceae, Ruminococcus, Faecalibacterium, Anaerostipes, and Prevotella [29]. However, the study of Chen et al. showed that Bacteroides were higher in NMDAR patients, contrasting the results of this study. There was an increased level of inflammatory cytokines like IFN-b, IFN-g, IL-3, TNF-a, etc., in the patient group and a decreased level in healthy controls which meant that these cytokines are positively related to the increased organisms listed above and this could predict a more severe disease and vice versa. The above-listed organisms in decreased abundance reduce the inflammation and are associated with less severe disease. The reason this dysbiosis leads to the pathogenesis of the disease is hypothesized to be due to molecular mimicry, which leads to cross-reaction to body components and an autoimmune attack on tissues. Higher bacterial diversity at admission was shown to have considerably better long-term outcomes and significantly decreased chances of relapse in patients while patients with lower bacterial diversity were more likely to develop relapse [30].

Gastrointestinal Dysbiosis and Brain Tumors

In a study by Li et al. on brain tumor patients, Fusobacteriota, Proteobacteria, Bacteroidetes, Escherichia /Shigella, Fusobacterium, Sutterella and *Ruminococcus gnavus* group were increased and Bifidobacterium or Lachnospira, Firmicutes, Actinobacteria and Parasutterella were decreased in disease group compared to controls. Fusobacteria and Proteobacteria showed a higher abundance in patients with malignant brain neoplasms than those with benign brain tumors [12]. In the benign brain neoplasm group, Bacteroidetes were especially increased. There was a higher abundance of Enterobacteriaceae and a lower abundance of Lachnospiraceae in the malignant group than in the benign group. In the malignant group, this study showed a higher abundance of Fusobacteria and Proteobacteria similar to the study by Chen et al. which revealed that Fusobacteria are known to promote tumor cell proliferation by influencing the activity of Regulatory T (Tregs) cells [30]. In this study, Sutterella was found to be increased in the tumor group, which is similar to the results of the study conducted by Kaakoush et al. in 2020 which showed that Sutterella reduces microbial diversity and promotes tumor progression. These bacteria in higher abundance groups, had an increase in cellular pathways leading to the accumulation of toxic substances while decreasing the number of pathways of normal homeostasis like biosynthetic reactions and energy production, etc. Fusobacteria promote tumor proliferation by affecting T regulatory cells and the process of autophagy. As such, this dysbiosis of organisms can be used as a non-invasive means to detect brain tumors at early stages [29,31].

Gastrointestinal Dysbiosis and Intracerebral Hemorrhage

In the study by Luo et al. involving patients with intracerebral hemorrhage (ICH) and coronary heart disease (CHD), Enterococcus, Parabacteroides, Lachnoclostridium, Acidaminococcus, Streptococcus, Blautia, Acidaminococcus, Alistipes, Hungatella, and *C. innocuum* group were increased in ICH group while Prevotella, Roseburia, and Faecalibacterium were depleted. It was found that ICH itself was the major cause of dysbiosis rather than other comorbidities like hypertension, diabetes, etc. In comparison with the control groups, the gut microbiota of the patients with ICH had increased microbial diversity, more abundance of opportunistic pathogens, and depletion of anaerobic bacteria. Enterococcus was correlated positively with the severity of ICH while Prevotella and Roseburia were negatively related to the severity and poor outcomes of ICH [13]. Similar to a study by Bousbia et al., Prevotella was linked with a reduced incidence of hospital-acquired pneumonia. Enterococcus was enriched in ICH patients who developed SAP [32]. This is in line with the study by Xia et al. which revealed that Enterococcus was abundant in SAP following acute ischemic stroke. Enterococcus and Parabacteroides could be used as biomarkers in the prediction of which patients with ICH develop SAP [33].

Xia et al. led a study on patients with ischemic stroke and found that Enterobacteriaceae and Parabacteroides had increased abundance in stroke patients having higher SDI and decreased abundance of Fecalibacterium, Clostridiaceae, and Lachnospira in such patients [15]. It correlates with the finding by Mancini et al. who showed that high Enterobacteriaceae at the beginning of transplant was a marker for the risk of sepsis and lower Lachnospiraceae (containing Lachnospira) was the only factor for increased risk of overall mortality in such patients. Thus, increased Enterobacteriaceae and lower Lachnospiraceae in patients having high SDI may be associated with exaggerated immune response and brain injury, and poor stroke outcome. It was found that SDI was positively related to patients’ stroke severity and poor early outcomes. It also demonstrated that SDI was an independent predictor of stroke severity and early unfavorable outcomes [34].

Gastrointestinal Dysbiosis and Neurocritical Illness in General

Xu et al. conducted a study on neuro-critically ill patients and found that Enterobacteriaceae, Porphyromonadaceae, Enterococcaceae, Verrucomicrobiaceae, Rikenellaceae, Proteobacteria, and Lactobacillaceae were increased and Firmicutes and Bacteroidetes were depleted in neurological intensive care unit (neuroICU) patients as compared to healthy controls. A clear increase in the growth of opportunistic microbiota was seen in neurocritical patients. This ICU-acquired dysbiosis was likely a consequence of multiple stressors in modern intensive care therapy like the utilization of multiple antibiotics, inotropic supports, enteral or parenteral feeding, proton pump inhibitors, analgesics, or sedative agents that impair intestinal motility and barrier function leading to a cascade of events observed thereafter. The abundance of intestinal Enterobacteriales and Enterobacteriaceae during the first week in the neuroICU was related with a significant increase in 180-day mortality in patients. Previous studies have shown Enterobacteriaceae to be one of the most detrimental pathogens to ICU patients. The increased abundance of Christensenellaceae and Erysipelotrichaceae can be used as potential risk indicators of mortality within 180 days of admission [14].

Gastrointestinal Dysbiosis in Other Neurological Diseases

The meta-analysis by Pammi et al. found that gut dysbiosis preceded the development of necrotizing enterocolitis and was characterized by increased abundance of Proteobacteria and decreased abundances of Firmicutes and Bacteroidetes. These findings are compatible with the results by Xu et al. that demonstrated a lower number of Firmicutes and Bacteroidetes in neurocritical patients [14,16,35]. Obese patients have more bacteria belonging to the phylum Bacteroides which is similar to findings by Li et al. which showed increased Bacteroides in brain tumor patients [12,36]. In patients suffering from depression, there is a decreased concentration of Bifidobacterium, Firmicutes, Faecalibacterium, Lactobacillus, and Ruminococcus, while higher concentrations of Prevotella, Bacteroides, and Proteobacteria. [36]. This is opposite to the findings by Luo et al. indicating Prevotella is reduced in patients with ICH. In Alzheimer's disease, there was a lower number of Firmicutes and Bifidobacterium and a higher abundance of Bacteroidetes similar to the changes in the brain tumor group in a study by Li et al. [12,13].

Limitations

Some authors mentioned bacterial phyla names while describing their findings while other authors used names of species while describing dysbiosis. If all the authors can report their findings by either using the name of bacterial species or their phyla, it would be beneficial for the consistency of this review. No study reported the quantitative number of bacteria colony-forming units (CFUs) while describing dysbiosis. Only qualitative terms like increased, higher, decreased, lower, etc., have been used. Although the control group was selected from the same region to minimize the bias, there can be some variation due to different eating habits of individuals, medication effects, etc. Gut flora also tends to change with age, so with different age groups in different studies, the results can be difficult to compare. Moreover, as the duration of the disease increases, and it becomes more severe, gut flora also gets affected. So, patients with chronic severe illness will have different flora as compared to the ones who have had milder versions of the disease.

Clinical recommendations

Gut microbiota should be assessed along with neuroimaging and behavioral testing for diagnosis of neurological or psychiatric disorders. Clinical trials should be conducted in humans to evaluate the efficacy of treatments targeting gut microbiota in the treatment of gut-brain disorders. In ICU patients, certain bacteria should be monitored as a decreased number of Bifidobacterium has been shown to improve prognosis in such patients. Probiotics in stroke patients can promote homeostasis in gut flora and can improve prognosis in these patients.

## Conclusions

In all neurocritical illnesses, dysbiosis has physiologic and pathologic effects that impact patient prognosis. In encephalitis patients, the levels of inflammatory cytokines, such as IFN-b, IFN-g, IL-3, and TNF-a, were directly correlated with levels of the previously listed bacteria and could reflect disease severity. Patients suffering from malignant brain tumors had increased amounts of Fusobacteria, Proteobacteria, and Enterobacteriaceae, while Lachnospiraceae decreased. In contrast, those patients with benign brain tumors had especially higher amounts of Bacteroidetes. As such, the specific dysbiosis could be used for both early detection and grading of tumor severity in a noninvasive manner. Similarly, in ischemic stroke patients, Enterobacteriaceae and Parabacteroides were linked to a higher SDI, while decreased amounts of Fecalibacterium, Clostridiaceae, and Lachnospira were linked to a higher SDI. Since SDI is an independent predictor of stroke severity and poor prognosis, dysbiosis can potentially be used as a prognostic indicator. Moreover, higher levels of Enterococci were found in both ICH and ischemic stroke patients who developed SAP. Since enterococcus was inversely correlated with IP-10 and directly correlated with IL-1RA, these cytokines could also be used as biomarkers of disease since a change in their levels (among others) was significantly associated with SAP.

We emphasize that gastrointestinal dysbiosis has a significant and profound effect on disease pathogenesis, severity, and prognosis in neuro-critically ill patients. We infer that neurocritical patients do not have much in common with respect to their dysbiotic microflora of the gut, rather they have disease-specific bacterial disproportion. Fluctuations in specific microbiota correlated with disease severity and prognosis. Moreover, the inhabiting population of dysbiotic organisms in neuro-critically ill patients were different in different diseases and there were no similarities in the composition of gut microbiota in these diseases. 
